# Expression of nodal signalling components in cycling human endometrium and in endometrial cancer

**DOI:** 10.1186/1477-7827-7-122

**Published:** 2009-10-29

**Authors:** Irene Papageorgiou, Peter K Nicholls, Fang Wang, Martin Lackmann, Yogeshwar Makanji, Lois A Salamonsen, David M Robertson, Craig A Harrison

**Affiliations:** 1 Prince Henry's Institute of Medical Research, 246 Clayton Road, Clayton, Vic 3168, Australia; 2 Department of Obstetrics and Gynecology, Monash University, Clayton, Vic 3168, Australia; 3 Department of Biochemistry and Molecular Biology, Monash University, Clayton, Vic 3168, Australia

## Abstract

**Background:**

The human endometrium is unique in its capacity to remodel constantly throughout adult reproductive life. Although the processes of tissue damage and breakdown in the endometrium have been well studied, little is known of how endometrial regeneration is achieved after menstruation. Nodal, a member of the transforming growth factor-beta superfamily, regulates the processes of pattern formation and differentiation that occur during early embryo development.

**Methods:**

In this study, the expression of Nodal, Cripto (co-receptor) and Lefty A (antagonist) was examined by RT-PCR and immunohistochemistry across the menstrual cycle and in endometrial carcinomas.

**Results:**

Nodal and Cripto were found to be expressed at high levels in both stromal and epithelial cells during the proliferative phase of the menstrual cycle. Although immunoreactivity for both proteins in surface and glandular epithelium was maintained at relatively steady-state levels across the cycle, their expression was significantly decreased within the stromal compartment by the mid-secretory phase. Lefty expression, as has previously been reported, was primarily restricted to glandular epithelium and surrounding stroma during the late secretory and menstrual phases. In line with recent studies that have shown that Nodal pathway activity is upregulated in many human cancers, we found that Nodal and Cripto immunoreactivity increased dramatically in the transition from histologic Grade 1 to histologic Grades 2 and 3 endometrial carcinomas. Strikingly, Lefty expression was low or absent in all cancer tissues.

**Conclusion:**

The expression of Nodal in normal and malignant endometrial cells that lack Lefty strongly supports an important role for this embryonic morphogen in the tissue remodelling events that occur across the menstrual cycle and in tumourogenesis.

## Background

Nodal, a member of the transforming growth factor-beta (TGF-β) superfamily, regulates the processes of pattern formation and differentiation that occur during early embryo development [1]. In particular, Nodal signalling is essential for mesoderm and endoderm induction, neural patterning and the specification of the primary body axes [1]. Nodal signals through activin type I (ALK4) and type II (ActRII or ActRIIB) serine-threonine kinase receptors [2]. However, unlike activin, Nodal lacks intrinsic affinity for ALK4 and ActRII/IIB, suggesting the requirement for a co-receptor to potentiate its actions [3]. Indeed, recent studies have shown that Nodal effects are dependent upon interactions with Cripto, a small cysteine-rich extracellular protein that is attached to the plasma membrane through a glycosyl phosphatidyl inositol linkage [1]. Cripto interacts with Nodal and ALK4, independently, and promotes the formation of a stable high affinity complex with activin type II receptors [4]. Phosphorylation of ALK4 within this complex initiates signaling via Smad2/Smad3 signal transducers [3]. The TGF-β signaling antagonist, Lefty, blocks Nodal actions by competing for access to the ligand binding site of Cripto [5].

Consistent with its crucial developmental function, *nodal *is first expressed throughout the embryonic ectoderm shortly after implantation (5.25 days post-coitum). Expression continues during the initial stages of primitive streak formation and is then rapidly down regulated as the streak elongates. Subsequently, *nodal *expression is detected in a small subset of node progenitors, and following the formation of the morphologically distinct node becomes restricted to the edges of the notochordal plate [1,6,7]. Until recently, Nodal expression was widely thought to be embryonically restricted [8]. However, several studies have shown that Nodal and its signalling partners are expressed at defined stages in a variety of adult tissues, including the lactating mammary gland and regenerating islet cells in the pancreas [9,10]. In addition, there is increasing evidence that Nodal pathway activity is upregulated in many human cancers. Hendrix and colleagues [11,12] have shown that expression of Nodal in metastatic melanomas and breast carcinomas is correlated with cancer progression, whereas pathway inhibition decreases cell invasiveness, colony formation and tumourigenicity.

Components of the Nodal signalling pathway have also been detected in human endometrium. Lefty A, which was originally designated endometrial bleeding associated factor (*ebaf*), is highly expressed in endometrium during the late secretory and menstrual phases, but is significantly reduced in proliferative, early and mid-secretory endometria [13,14]. Lefty A stimulates the production of several matrix metalloproteinases and may be a key local regulator of focal extracellular matrix breakdown in the cycling human endometrium [15]. Furthermore, dysregulated endometrial expression of Lefty is associated with infertility [14], and *in vivo *gene transfer of Lefty leads to implantation failure in mice [16]. Curiously, given Lefty's well-documented mechanism of action during vertebrate embryogenesis [5,17], the presence of Nodal and Cripto mRNA in human endometrium has only recently been established [18].

In the current study, RT-PCR and immunohistochemistry were utilised to examine the site- and menstrual cycle stage-specific expression of Nodal and Cripto in the human endometrium. As recent studies have suggested that increased Nodal signalling has a key role in melanoma cell plasticity and tumourgenicity, the expression profiles of Nodal, Cripto and Lefty were also examined in endometrial carcinomas. The expression of Nodal and Cripto in normal and malignant endometrial cells that lack Lefty strongly supports an important role for this embryonic morphogen in the endometrial remodelling events that occur across the menstrual cycle and in tumourogenesis.

## Methods

### Tissue Collection

Ethical approval was obtained from appropriate Institutional Ethics Committees for all tissue and sample collections. Written informed consent was obtained prior to tissue and sample collection from all subjects. Human endometrial biopsies were obtained from normal fertile women undergoing curettage following laparoscopic sterilization or assessment of tubal patency. Cycle stage was confirmed by histological dating, according to standard criteria [19]; thereafter samples were allocated to one of six groups: menstrual (days 1-4, *n *= 4), early proliferative (days 5-8, *n *= 4), mid proliferative (days 8-10, *n *= 4), late proliferative (days 11-13, *n *= 4), early secretory (days 14-17, *n *= 4), mid secretory (days 18-24, *n *= 4) and late secretory (days 25-28, *n *= 4). Endometrial biopsies were divided in two: one portion was fixed in 10% buffered formalin overnight, then washed three times in Tris-buffered saline (TBS, pH 7.6) before routine histological processing to paraffin blocks. The remainder was placed into RNA Later solution (Ambion, Austin, TX) before snap-freezing and stored at -80°C for subsequent RNA extraction. No RNA was extracted from mid proliferative tissue samples.

The collection, grading and preparation for immunohistochemistry of endometrial cancer biopsies has been described previously [20]. In the current study, Grade 1 (*n *= 4), Grade 2 (*n *= 4) and Grade 3 (*n *= 4) endometrial carcinomas were examined for the immunolocalisation of Nodal, Lefty and Cripto.

### RNA extraction, Reverse Transcription and Quantitative real-time PCR

Total RNA was extracted from endometrial samples from each menstrual cycle phase (*n *= 4 per stage: menstrual, early and late proliferative, early, mid and late secretory) using a total RNA extraction kit (Qiagen; Hildens, Germany) as previously described [21]. Contaminating DNA was removed using a DNA free kit (Ambion, Austin, Texas) for 25 min at 37°C after which reverse transcription was performed with 500 ng total RNA/sample using SuperScript-III (Invitrogen, Carlsbad, California) following the manufacturer's instructions, before being snap frozen and stored at -80°C. Quantitative real-time PCR analysis was then performed using the Roche Lightcycler 380 (Roche, Basel Switzerland) with the FastStart DNA Master SYBR-Green 1 system (Roche), or the Corbett Rotor-Gene 2000 (Corbett Lifesciences, Sydney, Australia) using universal SYBR-Green (Invitrogen). Oligonucleotide primer sequences were obtained from published sources [22], or were designed using Primer3 software (see Table 1), and were ordered from Sigma Genosys (Castle Hill, Australia). Relative quantification of Nodal, Cripto and Lefty mRNA expression across the cycle was determined using the 2^-ΔΔCT ^method [23] with β-actin as internal control. Full details of PCR amplification conditions are summarised in Table 1. After 38 cycles of PCR, a melting curve analysis was performed to monitor PCR product purity, and in initial experiments, the identity of the amplicons was confirmed by DNA sequencing.

**Table 1:** Primer-specific conditions used for quantitative PCR analysis

**Gene**	**Primer sequence (5'-3')**	**size (bp)**	**Mg**^2+^**(mM)**	**Anneal temp (°C)**	**Ex'sion time (s)**	**Read temp (°C)**^1^
Cripto	F AAGATGGCCCGCTTCTCTTACAGT	511	2	64	20	88
	R AAAGTGGTAGTACGTGCAGACGGT					
Nodal	F AGACATCATCCGCAGCCTACA	330	3	59.2	20	88
	R GTCCATCTGAAACCGCTCTAAG					
Lefty A	F GGGAATTGGGATACCTGGAT	206	3	62	25	80
	R CTAAATATGCACGGGCAAGG					
β-Actin	F CGAGCGCGGCTACAGCTT	500	2	64	14	85
	R TCATACTCCTGCTTGCTGATCC					

^1 ^The temperature (temp) at which the fluorescence of the PCR product was quantified during LightCycler analysis.

### Immunohistochemistry

Antibodies directed against Nodal (goat anti-mouse Nodal; R&D Systems, Minneapolis, MN), Lefty A (goat anti-human Lefty; Santa Cruz Biotechnology, Santa Cruz, CA) Cripto (produced by M. Lackmann), and the Cripto binding protein, glucose-regulated protein 78 (GRP78; goat anti-human GRP78; Santa Cruz Biotechnology) were used to localize the respective proteins to 5-μm sections of formalin-fixed paraffin-embedded endometrial tissue using standard immunohistochemical protocols. These antisera were selected because we showed that they were suitable for detecting human endometrial proteins or they had previously been used for Western blotting of human endometrial tissue [24,25]. Briefly, sections were dewaxed in histosol, dehydrated in ethanol, and washed in water. Antigen retrieval was achieved by microwaving the sections in 1 mM EDTA-NaOH (pH 8.0, Titriplex III; Merck, Darmstadt, Germany) for 10 min before allowing to cool and equilibrating in 0.1 M PBS, pH 7.4. The sections were blocked for 30 min in 10% normal serum, after which the primary antibody (4 μg/ml (Nodal); 1.7 μg/ml (Cripto): 1 μg/ml (Lefty); 0.25 μg/ml (GRP78)), was added and the sections incubated O/N at 4°C for Nodal and Cripto, or 1 h at room temperature for Lefty and GRP78. Following extensive washing in PBS, slides were incubated with secondary antibodies (1:200) for 30 min at room temperature (goat anti-mouse HRP (Dako, Victoria, Australia) for Cripto; donkey anti-sheep/goat HRP (Chemicon, Billerica, MA) for Nodal; biotinylated rabbit anti-goat for Lefty and GRP78 detection (Vector Laboratories; Burlingame, CA, USA). For Lefty and GRP78 detection, an additional 30 min room temperature incubation with Vectastain ABC amplification (Vector Laboratories, Burlingame, CA) was required. The reaction product was developed using 3,3' diaminobenzidine tetrahydrochloride (Dako). Sections were counterstained with a 1:10 dilution of Harris' hematoxylin (Sigma-Aldrich, St. Louis, MO), dehydrated in ethanol, cleared in histosol and mounted using DPX (Sigma-Aldrich). The control sections received preimmune IgG (mouse or goat; AbD Serotec, Oxford, UK) diluted appropriately, in place of the primary antibody.

The intensity of staining was scored for each tissue and each antibody on a scale of 0 (no staining) to 4 (maximal intense staining) in each of the cellular compartments; luminal epithelium (if available), glandular epithelium, stroma and vasculature. One whole section was evaluated per sample by two independent observers and agreed scores were recorded. Data for Nodal, Cripto and Lefty expression in glandular epithelium and stroma is shown. N = 4 tissues were assessed for each stage.

### Detection of Nodal in human uterine fluid

The collection of human uterine lavages (uterine washings) has been described previously [26]. Lavage fluid from two normally cycling women and a woman with endometrial cancer was concentrated 50-fold using Nanosep microconcentration devices with a 10 K cut-off (Pall Life Sciences, East Hills, NY). Reduced samples were analysed by SDS-PAGE and Western blotting. The mature and precursor forms of Nodal were identified using a rat monoclonal anti-Nodal antibody (R&D Systems, Minneapolis, MN).

### Statistical analysis

To determine if there were significant differences in mRNA expression levels for Nodal, Lefty A and Cripto at the different stages of the menstrual cycle, data were subjected to analysis of variance with Tukey's post hoc test. A value of P < 0.05 was considered statistically significant.

## Results

### Changes in gene expression of Nodal, Lefty and Cripto in the endometrium across the menstrual cycle

**Figure 1:**
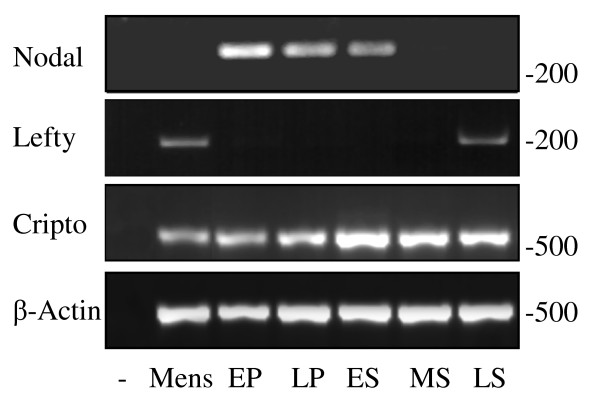
**Representative products from PCR reactions to amplify Nodal, Lefty and Cripto in endometrial samples collected at all stages of the menstrual cycle (n = 4/stage)**. A band corresponding to 330 bp was detected for Nodal, 206 bp for Lefty, and 511 bp for Cripto. PCR for β-actin (500 bp) on the same samples was performed to assess RNA loading. Mens = menstrual phase, EP = early proliferative, LP = late proliferative, ES = early secretory, MS = mid secretory, LS = late secretory.

Nodal, Lefty and Cripto mRNAs were expressed in non-pregnant endometrial samples, with cyclical variations evident (Fig. 1). Nodal mRNA expression was highest during the proliferative and early secretory phases before declining rapidly during the mid-secretory phase. This supports an earlier study, which showed Nodal mRNA was absent in mid-late secretory endometrium [27]. In contrast, Lefty mRNA expression was barely detectable during the proliferative and early secretory phases, but increased markedly during the late secretory phase and was maintained throughout menses. Cripto mRNA was expressed across the menstrual cycle, peaking during the secretory phase.

**Figure 2:**
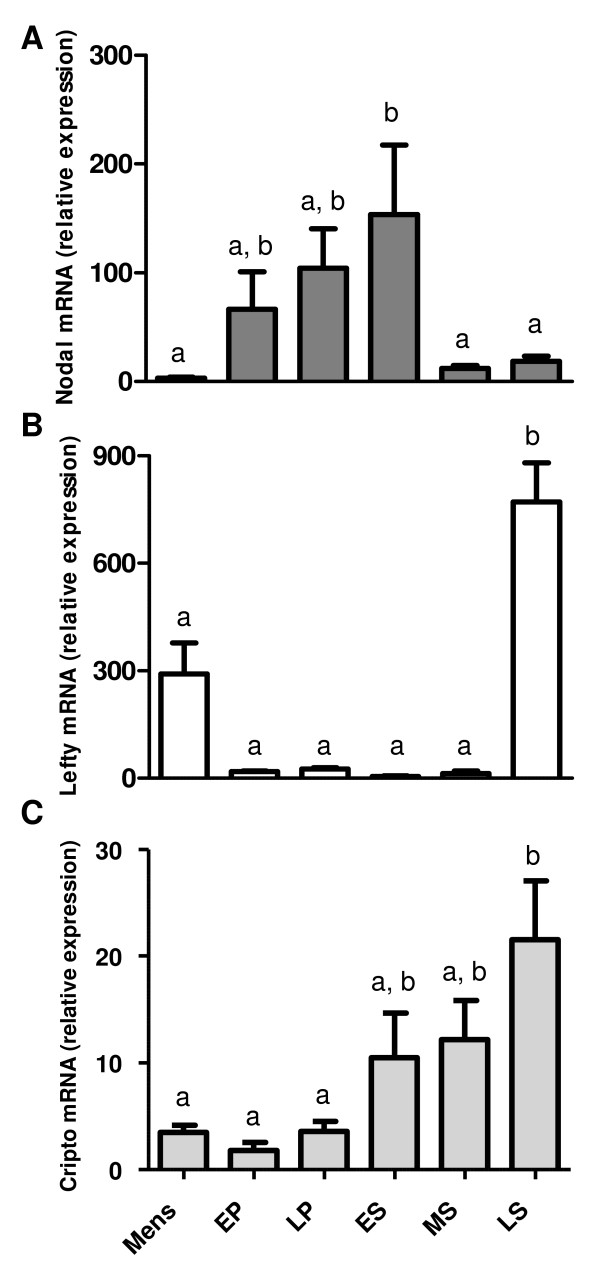
**Quantitation of Nodal, Lefty and Cripto mRNA expression patterns across the menstrual cycle by RT-PCR (normalized for β-actin mRNA expression)**. Histograms illustrating fluctuations in mRNA expression (mean values ± SEM) for Nodal, Lefty and Cripto across the menstrual cycle (Mens = menstrual phase, EP = early proliferative, LP = late proliferative, ES = early secretory, MS = mid secretory, LS = late secretory; *n *= 4 for each stage). Significantly elevated expression of Nodal mRNA was detected in the early secretory phase compared with the menstrual or mid/late secretory phases, while Lefty and Cripto mRNAs were significantly elevated in the late secretory phase only. Different letters indicate significant differences, P < 0.05.

To further investigate these cyclic variations, mRNA expression was quantitated using real-time PCR. The relative concentration of each PCR product was determined using the 2^-ΔΔCT ^method with β-actin mRNA expression as the internal control. Although this method does not allow for absolute quantitation of mRNA transcripts, the relative expression levels of individual genes can be compared between the different phases of the menstrual cycle. Nodal mRNA was significantly elevated (P < 0.05) during the early secretory phase of the menstrual cycle when compared with expression levels during the mid/late secretory and menstrual phases (Fig. 2A). Indeed, a 48-fold increase in Nodal mRNA expression was observed between the menstrual and early secretory phases. Strikingly, mRNA expression for the Nodal antagonist, Lefty, was nearly undetectable during the peak of Nodal expression, but rose dramatically (155-fold: P < 0.05) in the late secretory phase (Fig. 2B). Cripto mRNA expression increased marginally across the cycle, such that levels present in the late secretory phase were significantly (Fig. 2C; P < 0.05) higher than those observed during the menstrual and proliferative phases.

### Immunolocalization of Nodal, Lefty and Cripto in the endometrium throughout the menstrual cycle

**Figure 3:**
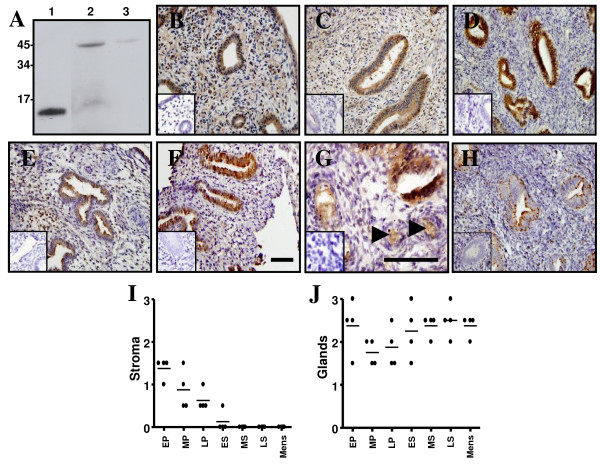
**Immunohistochemical localization of Nodal in endometrium across the menstrual cycle**. (A) Late secretory human endometrial tissue (*lanes 2 and 3*) was analysed by reducing SDS-PAGE and Western blot using a Nodal-specific antibody. A 13 kDa band representing mature Nodal monomer and a 40 kDa band representing Nodal precursor were detected in the absence of a blocking protein (*lane 2*). The inclusion of a blocking protein significantly reduced the intensity of the Nodal bands (*lane 3*). Recombinant Nodal (*lane 1*) was included as a positive control. The Nodal antibody was used to localize Nodal in early proliferative (B), late proliferative (C), early secretory (D & G), mid secretory (E) and menstrual phase (F) endometrium. Note the precipitous decrease in Nodal staining within the stromal compartment during the early/mid secretory phases. In menstrual tissue, Nodal expression coincided with the expression of its antagonist, Lefty (H). Negative controls for immunostaining by replacement of antisera with goat IgG at a matching concentration are shown in *insets*. *Arrowheads *(G) indicate specific Nodal staining in endothelial cells during the early secretory phase. *Bar*, 50 μm. Scale bar on (F) relates to panels (B-F & H), whereas scale bar on (G) relates to panel (G). Tissue sections were also scored according to Nodal staining intensity within endometrial stroma (I) and epithelial glands (J). Intensity of staining is shown as individual (filled circles) and mean scores (I & J).

Immunostaining of Nodal during the early proliferative phase of the menstrual cycle showed strong staining of stromal and epithelial cells (both glandular and luminal) (Fig. 3B). This pattern of staining was maintained across the proliferative phase (Fig. 3C), however, Nodal immunoreactivity was significantly decreased within the stromal compartment by the early secretory phase (Fig. 3D) and this was maintained through the late secretory and menstrual phases (Fig. 3E-F). In contrast, Nodal staining in both surface and glandular epithelium was sustained at relatively steady-state levels across the menstrual cycle (Fig. 3I). Additional staining was observed for Nodal in endothelial cells of spiral arterioles (Fig. 3G). The precipitous decrease in the stromal localization of Nodal during the secretory phase mirrors the decrease observed in Nodal mRNA at this time. Therefore, it is likely that stromal cells account for the majority of Nodal expression within the endometrium, although the possibility remains that other endometrial cells secrete Nodal into the stromal compartment.

Lefty A immunoreactivity within human endometrium has previously been shown to be restricted to glandular epithelium and surrounding stroma during the late secretory and menstrual phases [13] and our results support these findings (Fig. 3J and data not shown). Interestingly, within the glands Lefty expression was highly polarized with prominent apical and basal staining. Localized staining within the stroma was also evident with highest levels of Lefty expression observed in cells surrounding spiral arterioles (data not shown).

**Figure 4:**
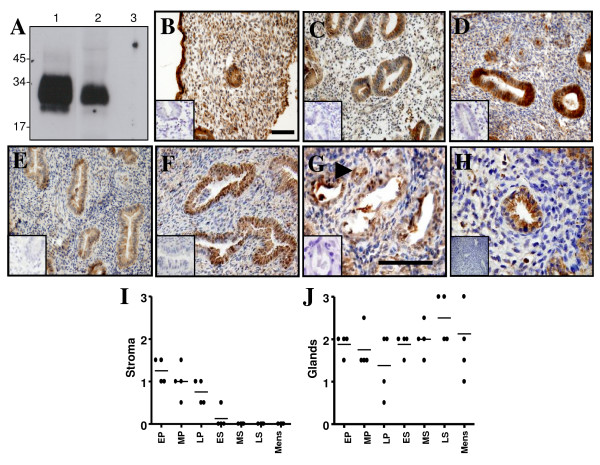
**Immunohistochemical localization of Cripto in endometrium across the menstrual cycle**. (A) Late secretory human endometrial tissue (*lanes 2 and 3*) was analysed by SDS-PAGE and Western blot using a Cripto-specific antibody. A 28 kDa band was detected in the absence (*lane 2*) but not the presence (*lane 3*) of a blocking protein. Recombinant Cripto (*lane 1*) was included as a positive control. The Cripto monoclonal antibody was used to localize Cripto in early proliferative (B), mid proliferative (C), early secretory (D), mid secretory (E & G) and menstrual phase (F) endometrium. Immunostaining for GRP78 in mid secretory endometrium (H) indicated that its expression coincided with that of its binding partner, Cripto. Negative controls for immunostaining by replacement of antisera with goat IgG at a matching concentration are shown in *insets*. *Arrowhead *(G) indicates specific Cripto staining in endothelial cells during the mid secretory phase. *Bar*, 50 μm. Scale bar on (B) relates to panels (B-F), whereas scale bar on (G) relates to panel (G & H). Tissue sections were also scored according to Cripto staining intensity within endometrial stroma (I) and epithelial glands (J). Intensity of staining is shown as individual (filled circles) and mean scores (H & I).

As expected, Cripto has a similar pattern of expression as its ligand, Nodal. During the proliferative phase, Cripto was strongly expressed in the stroma and the glandular and luminal epithelia (Fig. 4A &4B). Stromal Cripto staining decreased during the secretory phases (Fig. 4C &4D) and only low level staining was observed in the stromal compartment by the menstrual phase (Fig. 4E &4H). In contrast, Cripto immunoreactivity within the glandular and luminal epithelium increased across the cycle (Fig. 4I). Additional immunostaining was detected in endothelial cells of spiral arterioles (Fig. 4F). The recently identified Cripto binding protein, GRP78, has a similar expression pattern within the endometrium. GRP78 is strongly expressed in glandular and luminal epithelial cells and weakly expressed throughout the stroma (Fig. 4J). Together, Cripto and GRP78 are thought to form a complex at the cell surface and collaborate to mediate TGF-β superfamily signalling [28,29].

### Immunolocalization of Nodal, Cripto and Lefty in endometrial carcinomas

**Figure 5:**
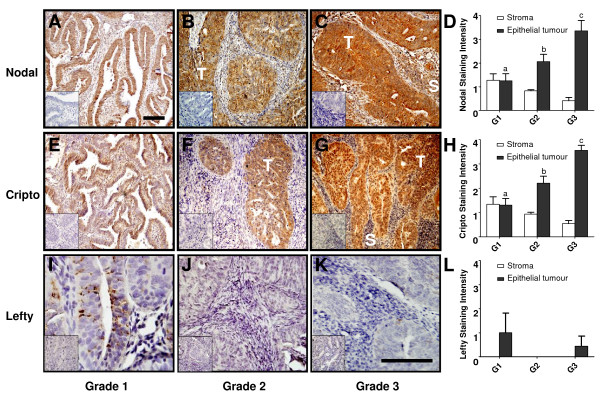
**Representative histochemical localization of Nodal (A-C), Cripto (E-G) and Lefty (I-K) in endometrial carcinomas of Grade 1 (A, E, I), Grade 2 (B, F, J) and Grade 3 (C, G, K)**. Tumour-associated (*T*) Nodal and Cripto staining increased in intensity with increasing tumour grade, whereas stromal (*S*) staining remains relatively unchanged. Negative controls for immunostaining by replacement of antisera with goat IgG at a matching concentration are shown in *insets*. *Bar*, 50 μm. Scale bar on (A) relates to panels (A-C & E-G), whereas scale bar on (K) relates to panels (I-K). Tissue sections were also scored according to Nodal (D), Cripto (H) and Lefty (L) staining intensity within endometrial stroma and tumour epithelial cells. Intensity of staining is shown as mean ± SD.

A recent study [12] showed that Nodal expression is positively associated with melanoma tumour progression: there is more Nodal protein in metastases than in primary tumours and none at all in normal skin. This study implicated Nodal as a diagnostic marker of disease progression and a target for the treatment of aggressive cancers such as melanomas [12]. Given that the components of the Nodal signaling pathway are present within the endometrium, we next assessed their expression patterns during the progression of endometrial cancer. Using immunohistochemistry, we found that Nodal expression in Grade 1 endometrial carcinomas is similar to that observed in the normal mid-proliferative endometrium (Fig. 5A): prominent staining in the glandular epithelial tumour cells and moderate staining in the stroma. In Grade 2 and Grade 3 endometrial carcinomas, Nodal staining increased dramatically and was primarily localized to the tumour cells, although less prominent tumour stroma staining was also evident (Fig. 5B &5C).

As in the normal endometrium, Cripto co-localized with Nodal in the endometrial carcinomas (Fig. 5D-F). In Grade 1 carcinomas, moderate Cripto staining was observed in both the glandular epithelial tumour cells and the surrounding stroma (Fig. 5D). Cripto staining intensity increased in the transition from histologic Grade 1 to histologic Grades 2 and 3 carcinomas. Strikingly, the expression of the Nodal antagonist, Lefty A, was reduced in Grade 1 carcinomas (Fig. 5G) compared to late secretory endometrium (Fig. 3J). Moreover, Lefty expression was absent in Grade 2 and 3 carcinomas (Fig. 5H-I); suggesting that Nodal signalling in these tumours would be unregulated.

### Nodal is secreted into the uterine lumen

**Figure 6:**
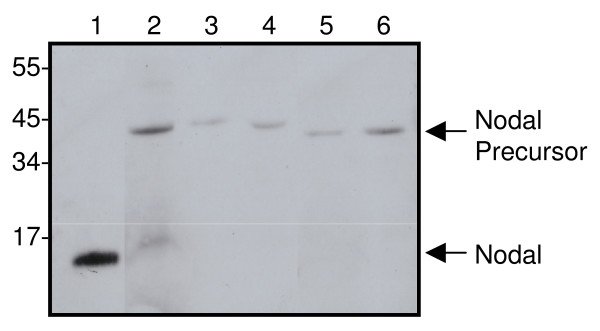
**Nodal Expression in uterine lavages**. Concentrated uterine lavage fluid from normal cycling women (*lanes 3 and *4) or a woman with endometrial cancer (*lane *5) was analysed by SDS-PAGE and Western blot using a Nodal-specific antibody. Recombinant Nodal (*lane 1*) and late secretory endometrial tissue (*lane 2*) were included as positive controls for the mature and precursor forms of Nodal, respectively. The size of molecular weight markers is indicated.

As the majority of glandular derived products are secreted apically, we examined uterine lavages for the presence of Nodal. Western blot analysis of concentrated lavage fluid indicated that the 50 kDa Nodal precursor was present in samples from both normal cycling women and women with endometrial cancer (Fig. 6). The Nodal precursor has previously been shown to be the major secreted form of the protein [30].

## Discussion

The human endometrium is divided into two layers: the functionalis, which comprises the upper two-thirds, and the basalis, which remains during and following menstruation and is thought to be the origin of a new functionalis in the subsequent cycle [31]. The restructuring of the functional layer is critical to the development of a tissue ready for implantation or, in the absence of a conceptus, for menstruation [32]. Endometrial repair and regeneration involves re-epithelialization (which occurs very rapidly even while menstruation is still in progress), angiogenesis and vessel remodeling, stromal and glandular epithelial cell proliferation, and extracellular matrix (ECM) deposition ([33]. Adult stem/progenitor cells likely underpin most aspects of this endometrial regeneration [31], however, the molecular and cellular mechanisms that mediate the regeneration process are still not well understood.

Given their established roles in wound healing, cell growth, ECM production and the maintenance of stem cell pluripotency [34-36], members of the TGF-β superfamily that signal via Smad2/3 (e.g. TGF-β1, TGF-β2, TGF-β3, activin A, activin B and Nodal) are likely to be important mediators of endometrial repair and regeneration. Indeed, endometrial repair is retarded in the absence of activin A [37]. In this study, we showed that Nodal, as well as its co-receptor, Cripto, are co-expressed in human endometrial tissue throughout the menstrual cycle. Immunohistochemistry localized Nodal and Cripto primarily to stromal and epithelial cells, although moderate staining was also observed in endothelial cells associated with spiral arterioles. Nodal protein levels were maintained in the glandular and luminal epithelium at relatively steady-state levels across the cycle, however, stromal localization was primarily restricted to the proliferative and early secretory phases. Cripto displayed a similar spatiotemporal localization to Nodal within the endometrium, although the amount of Cripto detected in glandular epithelium increased significantly during the late secretory phase of the cycle.

To be responsive to Nodal, a cell must express activin type I and type II receptors, in addition to Cripto. Jones *et al*. [21] have shown that endometrial stromal cells express each of the activin receptor subtypes (ALK4, ActRII and ActRIIB) with highest expression during the early secretory phase. Potential functions of the Nodal signalling pathway within the endometrial stromal compartment can be extrapolated from its roles in embryogenesis [1]. In early development, Nodal acts as a graded morphogen, instructing stem cells to adopt specific cell fates concurrent with proliferation and migration [8]. Similar actions during the proliferative phase of the menstrual cycle would ensure Nodal plays a key role in endometrial restoration.

Interestingly, activin receptors are not present in either surface or glandular epithelium at any stage of the cycle [21], suggesting that Nodal expressed in these cells cannot be functioning in a paracrine/autocrine manner. Indeed, as the majority of glandular derived products are secreted apically, it seemed likely that the endometrium must secrete Nodal into the uterine cavity. In support, we identified Nodal precursor in the uterine lavage fluid of both normal cycling women and women with endometrial carcinoma. Roles for endometrially derived Nodal could be similar to those proposed for activins, i.e. embryogenesis [38], steroidogenesis [39] and trophoblast differentiation [40]. In contrast to Nodal, Cripto within the epithelial compartment may be functional due to its ability to directly activate mitogen-activated protein kinase (MAPK) and phosphatidylinositol 3-kinase (PI3K)/Akt pathways via c-Src [9,41]. These actions of Cripto have led to its designation as a tumour growth factor and may be particularly relevant in endometrial carcinomas.

During embryogenesis, members of the Lefty subclass of TGF-β proteins act as extracellular antagonists of the Nodal signalling pathway [17]. Lefty blocks the formation of the Nodal receptor complex by binding to Nodal directly [17] or by interacting with Cripto [5]. Lefty expression is absolutely dependent upon Nodal function and within the embryo and developing tissues, such as the pancreas [10], the juxtaposition of these two factors limits their respective range of influence. It is interesting, therefore, that Nodal and Lefty have spatially and temporally distinct patterns of expression within the endometrium. The lack of Lefty expression in the proliferative phase of the menstrual cycle would ensure that the morphogenic actions of Nodal were not restricted at this time. In contrast, co-localization of Nodal, Cripto and Lefty within glandular and luminal epithelial cells during the late secretory and menstrual phases suggests that, at these times, Nodal signalling requires strict control. Indeed, the recently identified role for Lefty as a key local regulator of focal ECM breakdown in the cycling human endometrium [15] may derive from its ability to antagonise Nodal signalling.

Finally, recent studies have shown that expression of Nodal in metastatic melanomas and breast carcinomas is correlated with cancer progression, whereas pathway inhibition decreases cell invasiveness, colony formation and tumourigenicity [11,12]. These findings are consistent with the upregulation of Cripto that is observed in many epithelial cancers [41,42], and with the ability of Cripto to initiate several aspects of tumour progression, including increased proliferation, migration, invasion, angiogenesis, and epithelial-to-mesenchymal transition [29]. In the current study, we showed that Nodal and Cripto are expressed in normal endometrium and that their expression is dramatically upregulated in endometrial carcinomas.

## Conclusion

The expression of Nodal in normal and malignant endometrial cells that lack Lefty strongly supports an important role for this embryonic morphogen in the tissue remodelling events that occur across the menstrual cycle and in tumourogenesis. In addition, these findings identify Nodal and Cripto as diagnostic markers of endometrial cancer progression and, potentially, as molecular targets for the treatment of these aggressive tumours.

## List of abbreviations

TGFβ: transforming growth factor-beta; ALK4: activin type I receptor; ActRII: activin type II receptor; GRP78: glucose-regulated protein 78; ECM: extracellular matrix.

## Competing interests

The authors declare that they have no competing interests.

## Authors' contributions

IP participated in the immunohistochemistry and PCR studies and helped draft the manuscript. PN participated in the immunohistochemistry studies. FW participated in the PCR studies. ML produced the Cripto monoclonal antibodies. YM helped draft the manuscript and performed the statistical analysis. LS provided endometrial tissues and intellectual input. DR participated in the studies design. CH conceived of the study, and participated in its design and coordination and helped to draft the manuscript. All authors read and approved the final manuscript.
